# Celiac Artery Stenting to Facilitate Hepatic Yttrium-90 Radioembolization Therapy

**DOI:** 10.1155/2012/236732

**Published:** 2012-12-06

**Authors:** Murthy R. Chamarthy, Terence W. Hughes, Mohit Gupta, Josephina A. Vossen, Noel B. Velasco, Kenneth M. Zinn

**Affiliations:** Bridgeport Hospital, Yale New Haven Health System, 267 Grant Street, Bridgeport, CT 06610, USA

## Abstract

Radioembolization offers a novel way to treat the nonresectable, liver predominant hepatic malignancies with better tumor response and overall progression-free survival rates. Transarterial catheter-based radioembolization procedure involves the hepatic arterial administration of glass- or resin-based beta emitting Yttirum-90 microspheres. Safe delivery of the tumoricidal radiation dose requires careful angiogram planning and coil embolization to quantify lung shunting and prevent systemic toxicity, respectively. Diagnostic pretreatment angiogram also serves to identify the hepatic arterial variant anatomy and other coexisting pathologies that might require a different or alternative approach. We describe a complex case of celiac artery stenosis with tortuous pancreaticoduodenal arterial arcade precluding access to the right hepatic artery for performing radioembolization. Celiac artery stenting of the stenosis was performed to facilitate subsequent safe and successful Yttrium-90 microsphere radioembolization.

## 1. Introduction

**Figure 1:**
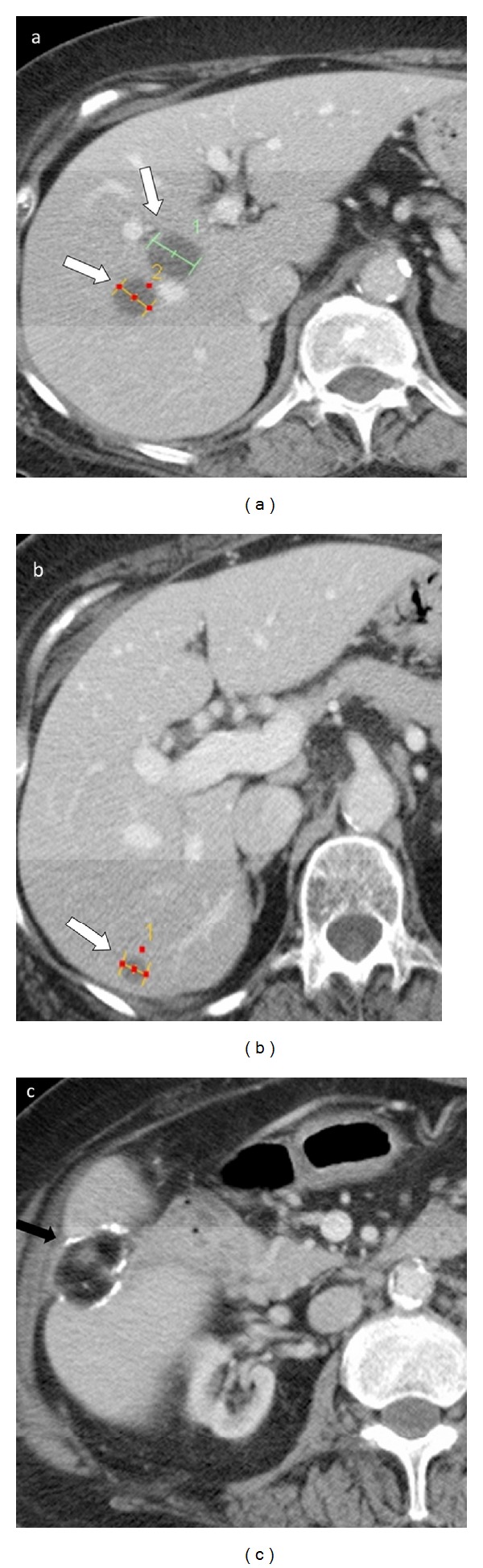
Contrast enhanced CT scan images demonstrate low attenuation right hepatic lobe lesions consistent with known metastatic disease (white arrows, (a) and (b)). Patient is status post partial segmental resection of the right hepatic lobe (black arrow, (c)).

A 74-year-old female with metastatic colorectal carcinoma to the right hepatic lobe (T_4_ N_2_ M_1_ at presentation) is status post colon and segment VI hepatic resection, and chemotherapy. She was referred for Yttrium-90 (^90^Y) radioembolization procedure for treatment of unresectable stable right hepatic lobe metastatic lesions (Figure 1). The liver and renal function tests and hematological indices were within normal range.

## 2. Mapping and Treatment Angiograms

**Figure 2:**
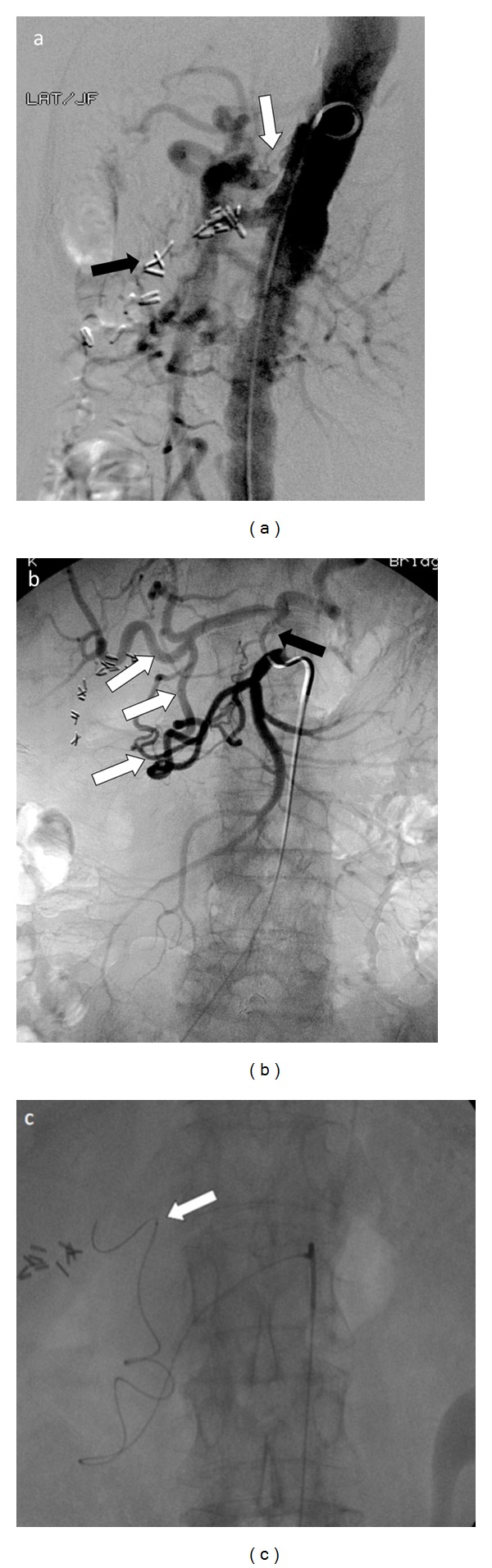
(a) Aortogram demonstrates a tight stenosis of the proximal celiac axis (white arrow) with mild poststenotic dilatation. In this case, the celiac artery stenosis was assumed to be related to atherosclerosis. The stenosis limits the access of the hepatic arterial branches for radioembolization purposes. Prior surgical clips related to partial right hepatic resection are noted (black arrow). (b) Superior mesenteric artery angiogram demonstrates significant hypertrophy and enlargement of the inferior pancreaticoduodenal arcade with opacification of gastroduodenal artery, and hepatic branches in a retrograde fashion (white arrows). There was a small collateral vessel which traveled from the superior mesenteric artery in a superior direction and opacified the very distal celiac axis and splenic artery (black arrow) likely representing the Arc of Buhler. (c) The right hepatic artery was not accessible through the tortuous pancreaticoduodenal collateral arcade (white arrow).

**Figure 3:**
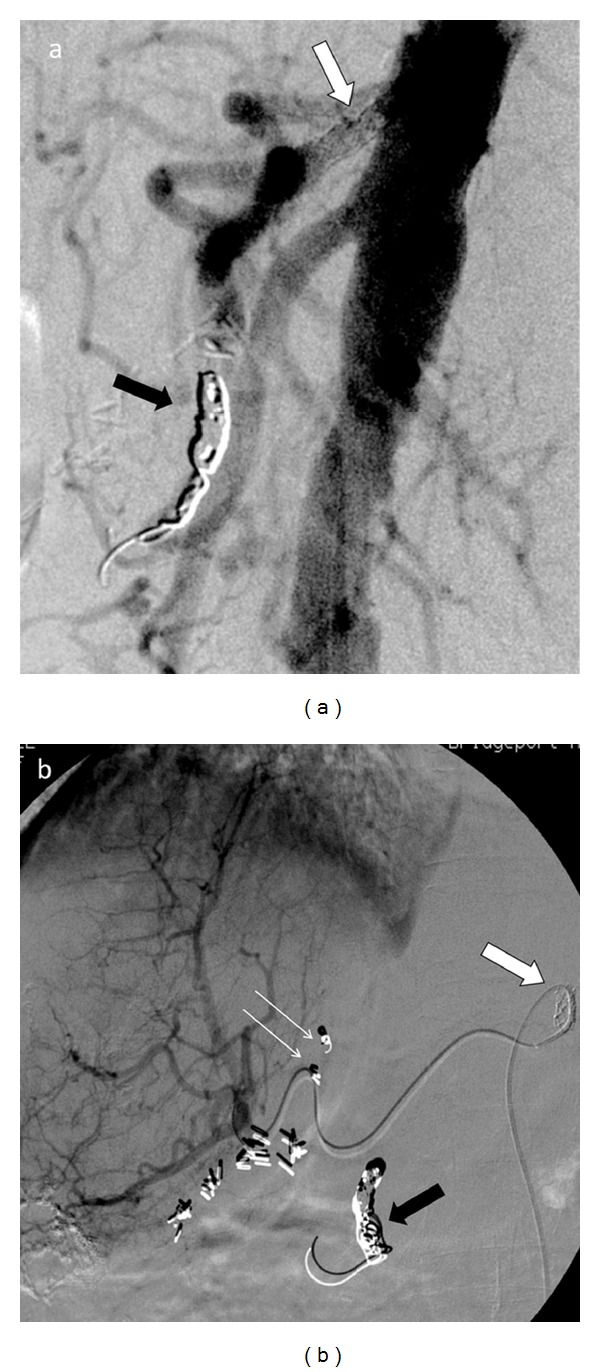
Treatment phase right hepatic angiogram images (a, b) demonstrate celiac artery stenting (6 × 15 mm Cordis chromium balloon expandable stent, white arrows) resulting in widely patent celiac axis with accessibility to the hepatic arteries. Coil embolization of gastroduodenal artery (two 10 mm × 10 cm tornado coils and two 8 mm × 4 cm coils, black arrows) prevents abnormal extrahepatic distribution. Additionally, a small accessory right hepatic branch was also coil embolized (two 3 × 2 mm platinum coils, white line arrows on (b)) to allow safer dose delivery and probable intrahepatic vascular flow redistribution [1, 2]. ^90^Y microspheres were injected within the right hepatic artery till end point without any immediate complications.

**Figure 4:**
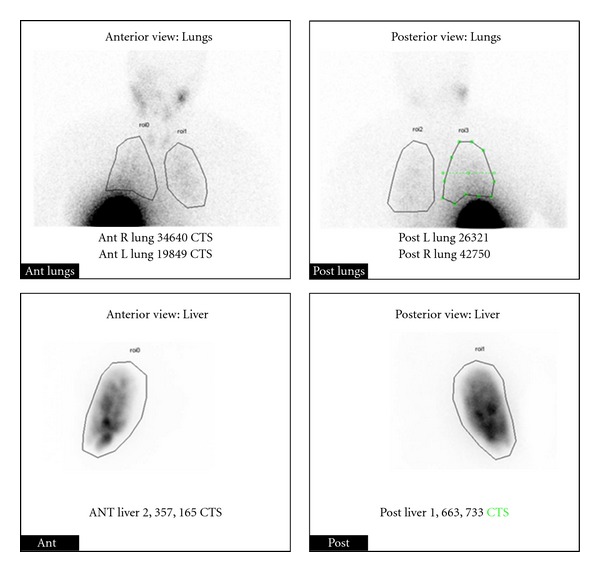
During the mapping angiogram phase, 5 mCi of technetium-99m labeled macroaggregated albumin was injected within the right hepatic artery to quantify the liver-lung shunt. The planar scintigraphic images demonstrate distribution of the radiotracer within the right lobe of the liver without any abnormal extrahepatic distribution. Regions of interest were drawn within the lungs and liver resulting in an estimated maximum lung shunting of 3%.

A planning or mapping angiogram was performed. The aortogram demonstrated a tight (approximately >90%) stenosis of the proximal celiac axis with mild poststenotic dilatation (Figure 2(a)). A superior mesenteric artery (SMA) angiogram demonstrated retrograde opacification of the hypertrophied pancreaticoduodenal arterial (PDA) arcade (Figure 2(b)). Despite using multiple microcatheter and guide wire combinations, catheterization of the right hepatic artery was unsuccessful given the extremely tortuous route precluding ^90^Y treatment. The only available remaining option for treatment was stenting of celiac artery to facilitate ^90^Y treatment. After appropriate discussion and consent, patient returned for additional mapping angiography, and a celiac artery stent was placed. Follow-up angiogram revealed widely patent celiac axis with no residual stenosis (Figure 3). Subsequently, coil embolization of the gastroduodenal artery (GDA) was performed via the stented celiac artery to prevent systemic radiotoxicity (Figure 3). Absence of abnormal extra hepatic radiotracer distribution and acceptable lung shunting was confirmed with intra-arterial administration of technetium-99m labeled macroaggregated albumin (^99m^Tc-MAA) within the right hepatic artery (Figure 4). 

**Figure 5:**
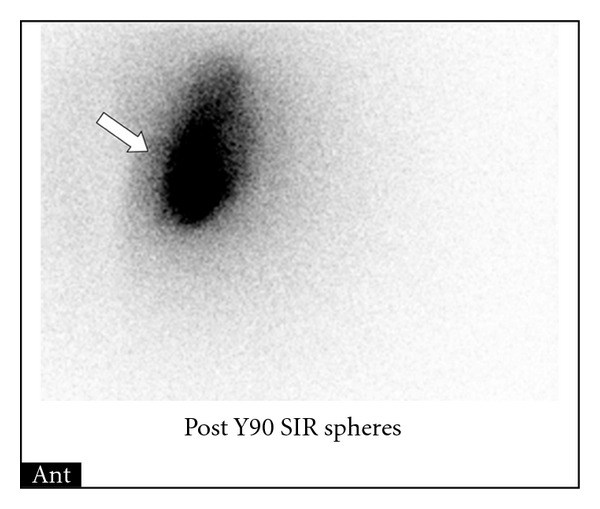
Posttherapy Bremsstrahlung image of the abdomen confirms proper delivery of microspheres to the targeted region of the right hepatic lobe (white arrow) without any abnormal extratumoral distribution. Safe and effective microsphere radioembolization with minimal to no radiotoxicity requires careful planning and preparation.

Approximately 27.8 mCi (1029 MBq) of ^90^Y microspheres (110% of calculated dose) was administered within the right hepatic artery through the stented celiac artery approach during the treatment phase utilizing the standard protocol till the end point. Bremsstrahlung images obtained after the ^90^Y radioembolization confirm proper delivery of microspheres to the targeted region without any abnormal extratumoral distribution (Figure 5). Patient tolerated the procedure without any immediate complications.

## 3. Discussion

Microsphere radioembolization therapy is currently approved for unresectable hepatic metastatic disease from colorectal cancer (SIR-Spheres, Sirtex Medical, Lane Cove, NSW, Australia) and unresectable hepatocellular carcinoma as a humanitarian device exemption (TheraSphere, MDS Nordion, Ottawa, ON, Canada) within the United States. ^90^Y microspheres are permanent biocompatible and non-biodegradable medical devices with a ^90^Y beta emitter within glass spheres or adsorbed onto resin spheres. Tumor cells receive the blood supply predominantly through the arterial system compared to the normal liver tissue which receives supply from the portal venous system. This preferential flow facilitates selective delivery of the radioactive microspheres to the tumor during transcatheter arterial therapies [1]. The selective and localized administration decreases the systemic toxicity and at the same time increases targeted delivery to tumor. Radioembolization provides the added benefit of beta radiation to adjacent tumor cells in the immediate vicinity [4]. Given these unique characteristics of the ^90^Y microsphere treatment, multiple studies reported improved tumor response and delayed time to progression compared to other treatment modalities [4–7]. 

The first step in the multidisciplinary approach is the planning or mapping angiogram phase that serves to confirm the normal or variant hepatic arterial anatomy and to coil embolize the GDA. Subsequently, Tc-99m MAA is injected to confirm lack of extrahepatic distribution and quantify the intrinsic tumoral lung shunting, which is used in conjunction with anatomical imaging volume and laboratory data for dose calculation. The treatment phase angiogram serves to reconfirm the arterial anatomy of the tumor, after which the ^90^Y microspheres are administered selectively up to the calculated dose or until flow stasis is achieved. A posttreatment Bremsstrahlung scan confirms dose delivery to tumor and absence of extrahepatic activity. These technical details and guidelines have been meticulously described previously [8–10]. Posttreatment complications are most often related to the abnormal systemic radioactive distribution and the systemic adverse effect profile has been proven to be superior compared to the traditional chemotherapy and radiation treatments [11, 12]. 

Arterial variants and abnormalities in the mesenteric and hepatic circulations might require alternate techniques for safe and effective radioembolization [13]. Celiac artery stenosis can result from atherosclerosis (as in the presented case), tumor invasion, localized inflammation, ligament compression, and rarely agenesis limiting the access to the hepatic branches through the celiac axis [14]. In these cases, the SMA usually provides a rich collateral supply predominantly through the PDA arcade, which can easily be utilized for selective access to the hepatic branches [8, 13, 15, 16]. An alternative approach would be celiac artery stenting that involves placement of a stent across the celiac stenosis with subsequent access to the hepatic artery branches. Celiac artery stenting was previously reported for treatment of variety of conditions including chemoembolization purposes [17–20]. 

Up to date, there have been a few case reports demonstrating the utilization of the SMA-inferior PDA approach for ^90^Y microsphere therapy in cases of celiac artery stenosis [8, 13]. The conceptual considerations of celiac artery stenting for microsphere radioembolization have been described [13]. There are no reported studies comparing the advantages and disadvantages of the two approaches described above. The relative choice of the approach is based on technical accessibility and individual preference. The clinical efficacy might be similar given the similar mechanism of localized delivery of the radioembolic microspheres. The retrograde PDA arcade approach has a theoretical risk of inadvertent radioembolization due to variation of flow patterns and possible technical difficulty to access hepatic artery branches in extremely tortuous cases. However, when technically possible, this retrograde approach obviates the need for an additional angiogram and GDA coil embolization. In our case, the SMA-PDA approach was not technically feasible and therefore celiac artery stenting provided the only route for selective access to the hepatic arteries. To our knowledge, this is the first reported case of celiac artery stenting to facilitate ^90^Y radioembolization treatment in English literature.

## 4. Conclusion

^90^Y microspheres radioembolization offers a unique opportunity to treat nonresectable, liver predominant hepatic malignancies with good tumor response, and overall progression free survival rates. Hepatic arterial variants and abnormalities such as arterial stenosis might limit access to the hepatic arterial branches supplying the tumor, and therefore, require alternate approaches or additional minimally invasive endovascular interventions. We report the use of stenting procedure in a case of celiac artery stenosis to facilitate hepatic ^90^Y microsphere radioembolization. This technique can be performed as a first line approach or in cases where the hepatic branches are not technically accessible through the conventional SMA-PDA arcade approach. Although the use of celiac axis stenting for ^90^Y microspheres radioembolization has been successful in our case, this approach should be evaluated further in a larger study population.

## Abbreviations

^99m^Tc-MAA: Technetium-99m macroaggregated albumin
^90^Y: Yttrium-90
GDA: Gastroduodenal artery
SMA: Superior mesenteric artery
PDA: Pancreaticoduodenal artery.
